# *Mycobacterium chimaera* Isolates from Heater–Cooler Units, United Kingdom

**DOI:** 10.3201/eid2307.170442

**Published:** 2017-07

**Authors:** Jessica Hedge, Theresa Lamagni, Ginny Moore, James Walker, Derrick Crook, Meera Chand

**Affiliations:** University of Oxford, Oxford, UK (J. Hedge, D. Crook);; Public Health England, London, UK (T. Lamagni, D. Crook, M. Chand);; Public Health England, Salisbury, UK (G. Moore, J. Walker);; Guy’s and St Thomas’ National Health Service Foundation Trust, London (M. Chand);; Imperial College London, London (M. Chand)

**Keywords:** tuberculosis and other mycobacteria, nontuberculous mycobacteria, equipment contamination, heater–cooler units, disease outbreaks, cross-infection, cardiac surgical procedures, United Kingdom, genome, bacteria, Mycobacterium chimaera

**To the Editor:** In their recent article, Svensson et al. provided results of an investigation into contamination of heater–cooler units (HCU) used in open-chest surgery with *Mycobacterium chimaera* in Denmark (*1*). We write to provide further information on the UK isolates included in their study.

The authors performed whole-genome sequencing (WGS) of 5 isolates sampled from HCUs in hospitals in Denmark, 1 from a Maquet HCU and 4 from Sorin 3T HCUs. They compared these sequences with publicly available WGS data for 31 *M. chimaera* isolates sampled in the United Kingdom, United States, and Ireland and reported, “*M. chimaera* sequences from the UK HCU water samples were genetically nearly identical to the US and Denmark isolates” (*1*). Without knowledge of the source of the 8 UK HCU isolates, and given the high genetic similarity found between Sorin 3T HCU isolates, the authors concluded that the UK isolates probably originated from Sorin 3T HCUs (*1*).

The WGS data for the UK isolates were generated as part of our investigation into the *M. chimaera* outbreak in the United Kingdom, which included national case finding, aerobiological investigations, environmental sampling, and phylogenetic analysis (*2*). We performed WGS for 246 *M. chimaera* isolates, including multiple isolates from 15 cardiothoracic surgery patients, 143 control patients without history of cardiothoracic surgery, and 11 HCUs. We found close phylogenetic clustering of isolates from cardiothoracic patients and HCUs. All HCU isolates for which WGS data were generated originated from Sorin 3T HCUs, including the 8 UK isolates analyzed by Svensson et al., confirming their inference of a shared origin. We have added this information to the corresponding US National Center for Biotechnology Information Short Read Archive BioProject (accession no. PRJNA324238). 

Our investigation also identified a mechanism of aerosol release through controlled laboratory assessment of a decommissioned HCU. Nationwide risk quantification identified a low but increasing risk of *M. chimaera* infection. A UK-wide patient notification exercise is underway (*3**,**4*).
